# Effect of metformin on all-cause and cardiovascular mortality in patients with coronary artery diseases: a systematic review and an updated meta-analysis

**DOI:** 10.1186/s12933-019-0900-7

**Published:** 2019-07-30

**Authors:** Yechen Han, Hongzhi Xie, Yongtai Liu, Peng Gao, Xufei Yang, Zhujun Shen

**Affiliations:** 1 Department of Cardiology, Peking Union Medical College Hospital, Chinese Academy of Medical Sciences and Peking Union Medical College, Beijing, 100730 China; 2 Peking Union Medical College and Chinese Academy of Medical Sciences, Beijing, 100005 China

**Keywords:** Coronary artery disease, Diabetes, Meta-analysis, Systemic review

## Abstract

**Background:**

Metformin is the most widely prescribed drug to lower glucose and has a definitive effect on the cardiovascular system. The goal of this systematic review and meta-analysis is to assess the effects of metformin on mortality and cardiac function among patients with coronary artery disease (CAD).

**Methods:**

Relevant studies reported before October 2018 was retrieved from databases including PubMed, EMBASE, Cochrane Library and Web of Science. Hazard ratio (HR) was calculated to evaluate the all-cause mortality, cardiovascular mortality and incidence of cardiovascular events (CV events), to figure out the level of left ventricular ejection fraction (LVEF), creatine kinase MB (CK-MB), type B natriuretic peptide (BNP) and to compare the average level of low density lipoprotein (LDL).

**Results:**

In this meta-analysis were included 40 studies comprising 1,066,408 patients. The cardiovascular mortality, all-cause mortality and incidence of CV events were lowered to adjusted HR (aHR) = 0.81, aHR = 0.67 and aHR = 0. 83 respectively after the patients with CAD were given metformin. Subgroup analysis showed that metformin reduced all-cause mortality in myocardial infarction (MI) (aHR = 0.79) and heart failure (HF) patients (aHR = 0.84), the incidence of CV events in HF (aHR = 0.83) and type II diabetes mellitus (T2DM) patients (aHR = 0.83), but had no significant effect on MI (aHR = 0.87) and non-T2DM patients (aHR = 0.92). Metformin is superior to sulphonylurea (aHR = 0.81) in effects on lowering the incidence of CV events and in effects on patients who don’t use medication. The CK-MB level in the metformin group was lower than that in the control group standard mean difference (SMD) = − 0.11). There was no significant evidence that metformin altered LVEF (MD = 2.91), BNP (MD = − 0.02) and LDL (MD = − 0.08).

**Conclusion:**

Metformin reduces cardiovascular mortality, all-cause mortality and CV events in CAD patients. For MI patients and CAD patients without T2DM, metformin has no significant effect of reducing the incidence of CV events. Metformin has a better effect of reducing the incidence of CV events than sulfonylureas.

## Background

Coronary artery disease (CAD) is the most common cardiovascular disease. It is a major cause of death and permanent disability and carries heavy economic and social costs due to its impaired functioning. It is estimated that up to 23.3 million people will die of cardiovascular disease by 2030 [1]. CAD posed challenges to developed countries as well as to developing countries. With the aging of the global population, CAD has become a major public health problem that seriously threatens human life and health [2, 3].

Metformin, as a biguanide derivative (dimethylbiguanide), has always been the primary drug in hypoglycemic treatment of patients with type 2 diabetes mellitus (T2DM) since its introduction in 1957 [4]. Studies have found that metformin not only has a hypoglycemic effect, but also has a protective effect on various diseases such as kidney cancer [5–7], pancreatic cancer, periodontal disease. Recent studies have also found that metformin has a positive effect on cardiovascular protection [8–12]. Metformin also lowers risk factors for cardiovascular disease such as blood fats [13–15], body weight and blood pressure. Compared with insulin and/or oral hypoglycemic agents (except metformin), metformin reduces the risk of all-cause mortality and the incidence of cardiovascular disease [16, 17], infection, or acidosis. Metformin can reduce the incidence of myocardial infarction (MI) in newly diagnosed obese diabetic patients [18]. Similarly, in animal models of myocardial infarction, metformin can effectively limit ischemia–reperfusion injury and reduce the infarct area, which is also shown in non-diabetic animals. Some placebo-controlled trials of metformin even support findings that incorporate cardiovascular endpoints [19]. However, the effect of metformin on cardiovascular disease, especially coronary heart disease, remains controversial. Therefore, it is necessary to provide these data to equip patients with treatment guidelines and prescribing decisions.

Therefore, in this changing context, it seems timely to review the evidence of effects of metformin in preventing and improving cardiovascular disease. So, the goal of this systematic review and meta-analysis was to assess the effects of metformin on cardiovascular mortality, all-cause mortality, cardiac function and low density lipoprotein (LDL) levels in patients with CAD.

## Methods

This study was reported in accordance with the PRISMA statement for reporting systematic reviews and meta-analysis [20].

### Search strategy

A systematic literature search of the PubMed, EMBASE, Cochrane Library and Web of Science databases was conducted by two study investigators independently. The cutoff date of the search was October 31, 2019. The following free text or MeSH terms were used in searching: “metformin” combined with “coronary heart disease” or “CHD” or “myocardial infarction” or “myocardial ischemia” or “cardiovascular disease” or “cardiovascular mortality” or “coronary artery disease” or “CAD” or “heart failure” or “HF” or “CHF”. The search was restricted to human studies. The titles and abstracts of studies identified in the search were independently reviewed by the two authors to exclude studies that were not meaningful to our research. References to identified studies were also retrieved to identify studies that may be eligible. The scope of inclusion was not limited by the language of publication.

### Selection criteria

Eligible patients: patients with CAD, and patients with other age-related comorbidities were not excluded.

Eligible interventions: in our study, patients in the intervention group had been treated by metformin for a period of time.

Eligible controls: no medication or drugs other than metformin were used in the control groups.

Primary outcome: cardiovascular events (CV events), defined as recurrent MI, heart failure (HF), recurrent angina, malignant arrhythmia, cardiogenic death), cardiovascular mortality and cardiac function.

Two authors independently extracted data from selected studies, one author use a predefined data extraction sheet to extract data from each of the included studies, the second author independently reviewed the data to ensure accuracy.

### Assessment of risk of bias

We used the Newcastle–Ottawa Scale (NOS) for assessing the quality of cohort studies and case–control studies based on three categories and eight items. The NOS uses a star rating system (semi-quantitative) where a maximum of nine stars can be awarded in assessing quality of studies.

### Data extraction and analysis

The analysis was carried out from three perspectives and six indexes. Firstly, HR was calculated between effects of metformin and non-metformin on cardiovascular mortality, all-cause mortality and incidence of CV events. Secondly, three cardiac function indexes including LVEF, CK-MB and BNP were tested, each of which got the mean value or mean standard differences pooled in metformin and non-metformin groups. Thirdly, mean difference of LDL level was calculated between metformin and non-metformin groups.

### Statistical methods

Review Manager 5.3 software from the Cochrane Collaboration (London, United Kingdom) was used to estimate the pooled effect size, the inverse variance approach was used to pool HR and SMD and draw the forest plot. *P* values for all comparisons were two-tailed and *P* < 0.5 of all tests was considered statistically significant except for heterogeneity. *I*^2^ values and Q statistic were used to evaluate heterogeneity across studies. Statistically significant heterogeneity was present at the *P* < 0.1. The random-effects model was used to calculate the pooled effect values where significant heterogeneity was present. Otherwise the fixed effects model was used.

## Results

### Study selection and characteristics

**Fig. 1:**
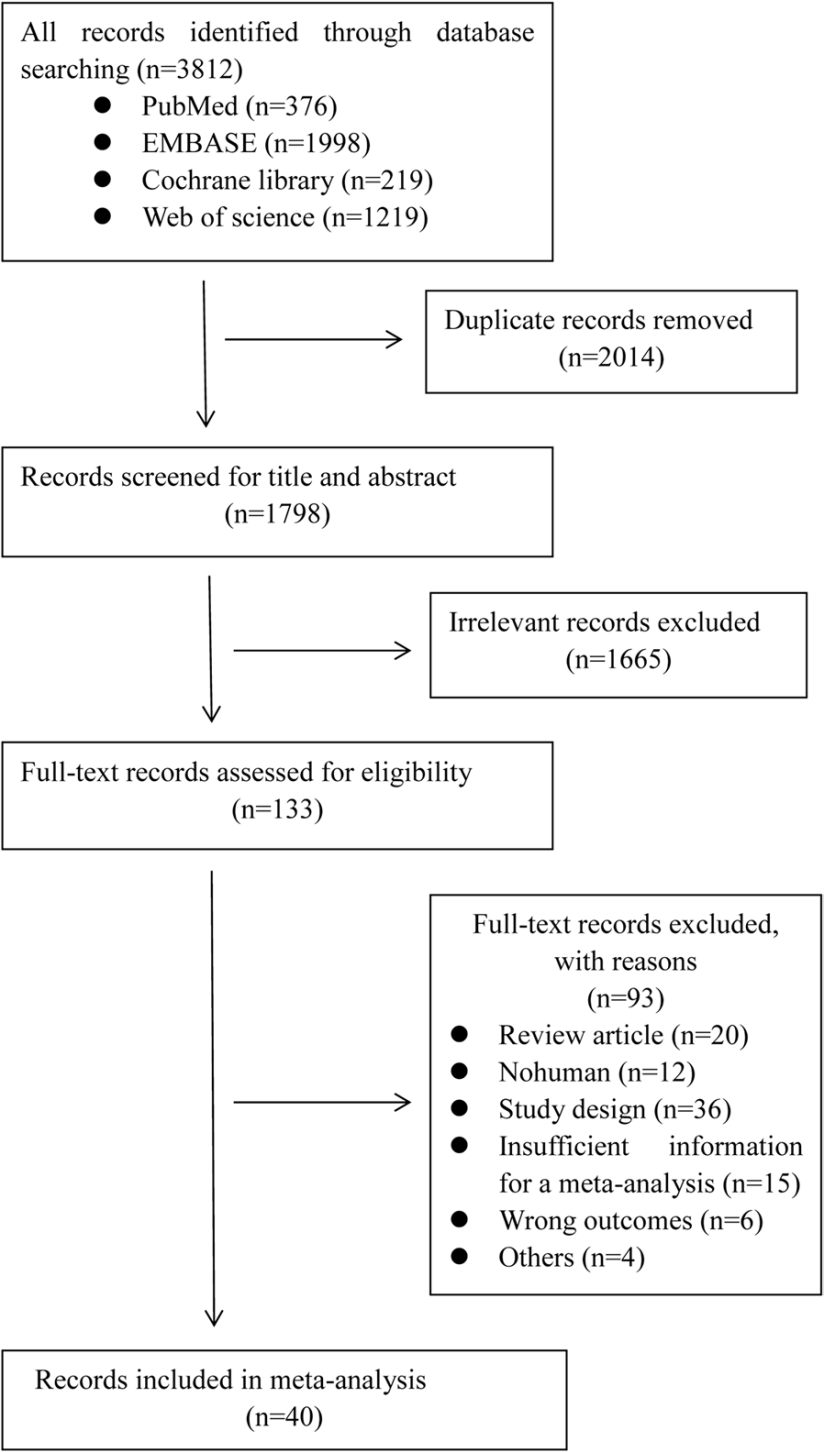
Flowchart of study selection

The flow diagram for retrieval and selection of studies is shown in Fig. 1. The comprehensive search identified 3812 articles, 2014 ones were excluded after screening title and abstract, 1665 ones were excluded after the full-text selection. We downloaded 133 articles, manually read the full text and finally decided to include 40 articles. 40 clinical trials enrolled 1,066,408 participants treated with metformin or allocated in control group, returned a lot of data that were consistent with our pre-defined outcomes, all of which were included in this meta-analysis. Among the included studies, 15 studies were of randomized controlled trials, 22 were retrospective cohort studies and 3 were case–control studies. Detailed baseline characteristics and quality of each study assessed according to the NOS were presented in Table 1.

**Table 1:** Characteristics of included studies

Author	Year	Study design	Gender male %	Age	Sample size	Follow-up (y)	Country	Patient	The NOS for assessing the quality of studies		
									Selection	Comparability	Exposure
Abualsuod [21]	2015	Retrospective cohort	52%	60.42 (13.36)	720	1	USA	MI + T2DM	☆☆☆	☆	☆☆☆
Al Ali [22]	2016	RCT	81%	57.9 (11.4)	237	0.3	Netherlands	MI-T2DM	☆☆☆☆	☆☆	☆☆
Basnet [23]	2015	Case control	65%	–	274	0.0	USA	MI + T2DM	☆☆☆	☆	☆☆
Chen [24]	2016	Retrospective cohort	53%	52.53 (10.07)	179,742	0.3	Canada	T2DM	☆☆☆	☆	☆☆
Duncan [25]	2007	Retrospective cohort	76%	65 (58–72)	1284	0.0	USA	CVD + T2DM	☆☆☆☆	☆	☆☆
Eppinga [26]	2016	RCT	75%	58.80 (11.82)	371	0.3	Netherlands	MI-T2DM	☆☆☆☆	☆☆	☆☆☆
Evans [27]	2006	Retrospective cohort	51%	60.2	7967	5.0	UK	T2DM	☆☆☆☆	☆☆	☆☆☆
Facila [28]	2017	RCT	56%	71 (10)	835	2.4	Spain	HF + T2DM	☆☆☆	☆☆	☆☆☆
Fung [29]	2015	Retrospective cohort	40%	61.70 (10.75)	11,293	5.0	China	T2DM	☆☆☆☆	☆	☆☆☆
Hartman [30]	2017	RCT	–	–	379	2.0	Netherlands	MI-T2DM	☆☆☆☆	☆☆	☆☆☆
Hong [31]	2013	RCT	78%	62.8 (8.5)	304	5.0	China	CVD + T2DM	☆☆☆☆	☆☆	☆☆☆
Johnson [32]	2005	Retrospective cohort	52%	64.3 (12.4)	4142	9.0	Canada	T2DM	☆☆☆	☆	☆☆☆
Jong [16]	2019	Retrospective cohort	74%	65.9 (10.8)	636	2.0	Taiwan	MI + T2DM	☆☆☆	☆	☆☆
Kitao [33]	2017	RCT	65%	60 (20–74)	96	0.3	Japan	T2DM-CVD	☆☆☆☆	☆☆	☆☆
Kooy [34]	2009	RCT	41%	64 (10)	390	4.0	Netherlands	T2DM	☆☆☆☆	☆☆	☆☆☆
Kruszelnicka [35]	2015	Case control	60%	67 (8)	70	0.0	Poland	CVD + T2DM	☆☆☆	☆	☆☆
Lexis [36]	2012	RCT	–	–	350	0.8–10.7	Netherlands	MI-T2DM	☆☆☆☆	☆☆	☆☆☆
Lexis [37]	2014	Retrospective cohort	64%	66 (12)	3948	0.3	Netherlands	MI + T2DM	☆☆☆	☆	☆
Lexis [38]	2015	RCT	77%	58.1 (11.9)	346	0.4	Netherlands	MI-T2DM	☆☆☆☆	☆☆	☆☆
Lexis [39]	2014a	RCT	75%	58.7 (11.8)	379	0.3	Netherlands	MI-T2DM	☆☆☆☆	☆☆	☆☆
Li [40]	2014	RCT	68%	62.4 (11.0)	152	1.0	China	T2DM	☆☆☆☆	☆	☆☆
Liu [41]	2016	Retrospective cohort	44%	60.7	272,149	7.4	USA	T2DM	☆☆	☆	☆
Liu [42]	2017	RCT	47%	59 (17)	60	0.5	China	CVD + T2DM	☆☆☆	☆	☆
Morgan [43]	2014	Retrospective cohort	36%	66.6 (10.4)	5208	0.0	UK	T2DM	☆☆☆☆	☆☆	☆☆☆
Pantalone [44]	2009	Retrospective cohort	42%	56.8 (13.9)	20,450	n	USA	T2DM-CVD	☆☆☆	☆	☆☆☆
Preiss [45]	2014	RCT	81%	63 (8)	173	1.5	UK	CVD-T2DM	☆☆☆☆	☆☆	☆☆☆
Rachmani [46]	2002	RCT	53%	65 (4)	393	4.0	Israel	T2DM	☆☆	☆☆	☆☆
Raee [47]	2017	Retrospective cohort	42%	55 (11.1)	717	13.6	Iran	T2DM	☆☆☆☆	☆	☆☆
Retwiński [48]	2018	Retrospective cohort	70%	64.5 (10.5)	1030	1.0	Poland	HF + T2DM	☆☆☆☆	☆	☆☆☆
Romero [49]	2013	Retrospective cohort	47%	70.5 (7.0)	1184	9.0	Spain	HF + T2DM	☆☆☆☆	☆	☆☆☆
Roumie [50]	2012	Retrospective cohort	97%	65 (57–74)	161,296	5.0	USA	T2DM	☆☆☆☆	☆☆	☆☆☆
Roumie [51]	2017	Retrospective cohort	97%	66 (57–75)	131,972	7.5	USA	T2DM	☆☆☆☆	☆☆	☆☆☆
Roussel [52]	2010	Retrospective cohort	66%	67.1 (9.3)	19,691	2.0	France	T2DM	☆☆☆☆	☆	☆☆☆
Scheller [53]	2014	Retrospective cohort	52%	59.0 (15.2)	84,756	4.0	Denmark	T2DM	☆☆☆☆	☆☆	☆☆☆
Schramm [54]	2011	Retrospective cohort	51%	52.5 (14.0)	110,374	9.0	Denmark	T2DM	☆☆☆☆	☆	☆☆☆
Shah [55]	2010	Retrospective cohort	79%	56 (11)	131	2.0	USA	HF + T2DM	☆☆☆	☆	☆☆☆
Sillars [56]	2010	Retrospective cohort	44%	60.6 (11.9)	1271	15.0	Australia	T2DM	☆☆☆	☆	☆☆☆
Wang [57]	2017	Retrospective cohort	–	72.49 (5.15)	41,204	8.0	USA	T2DM	☆☆☆	☆	☆☆☆
Wong [58]	2012	RCT	90%	64 (8)	62	0.3	UK	HF-T2DM	☆☆☆	☆☆	☆☆☆
Zeller [59]	2016	Case control	76%	61 (11)	372	0.0	France	MI + T2DM	☆☆☆	☆	☆☆
Total studies	40				1,066,408						

### Association of metformin with mortality

**Fig. 2:**
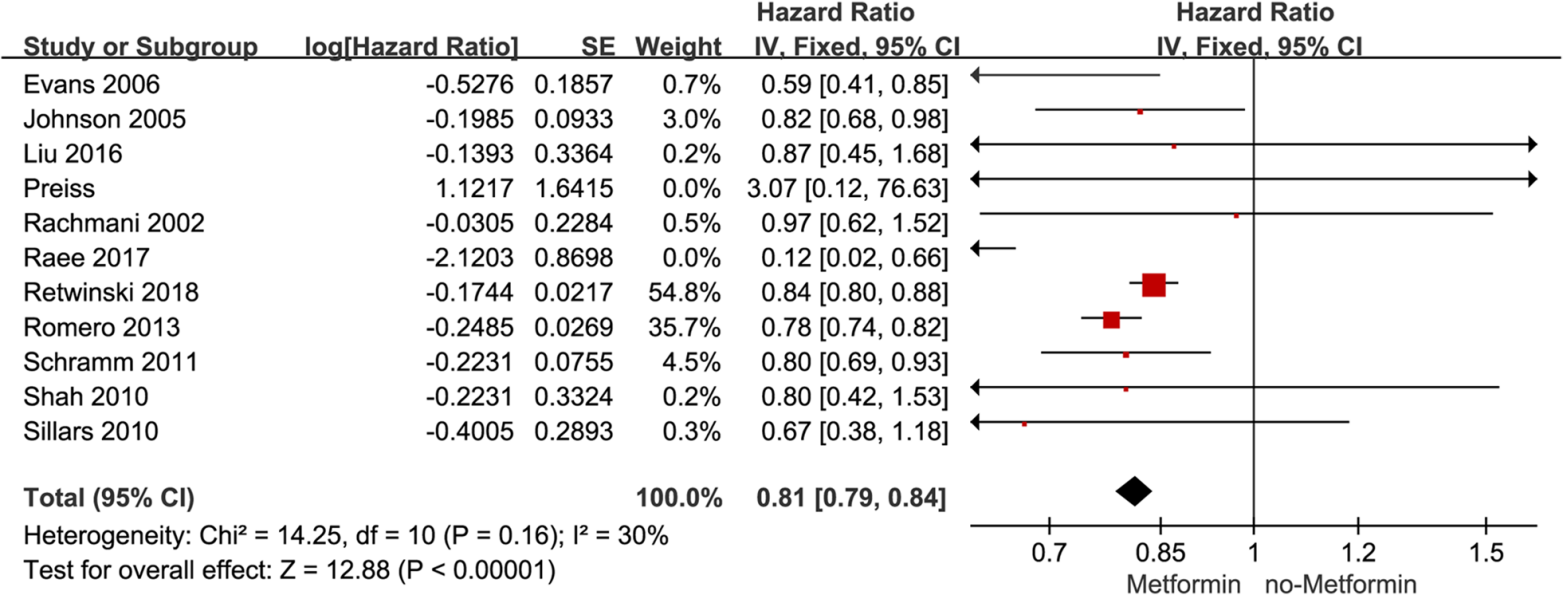
Forest plot of hazard ratio of cardiovascular mortality among patients with metformin therapy vs no-metformin therapy

Firstly, we investigated the association between metformin and cardiovascular mortality. Out of included 11 studies only one study Preiss 2014 presented HR > 1 [45], ten studies presented HR of cardiovascular mortality between metformin and non-metformin less than 1 (HR < 1), the adjusted hazard ratio (aHR) is 0.81 95% confidence interval (CI) (0.79, 0.84), (*P* < 0.00001), heterogeneity *I*^2^ is 30% (Fig. 2). It was found that metformin had a significant effect on lowering cardiovascular mortality.

**Fig. 3:**
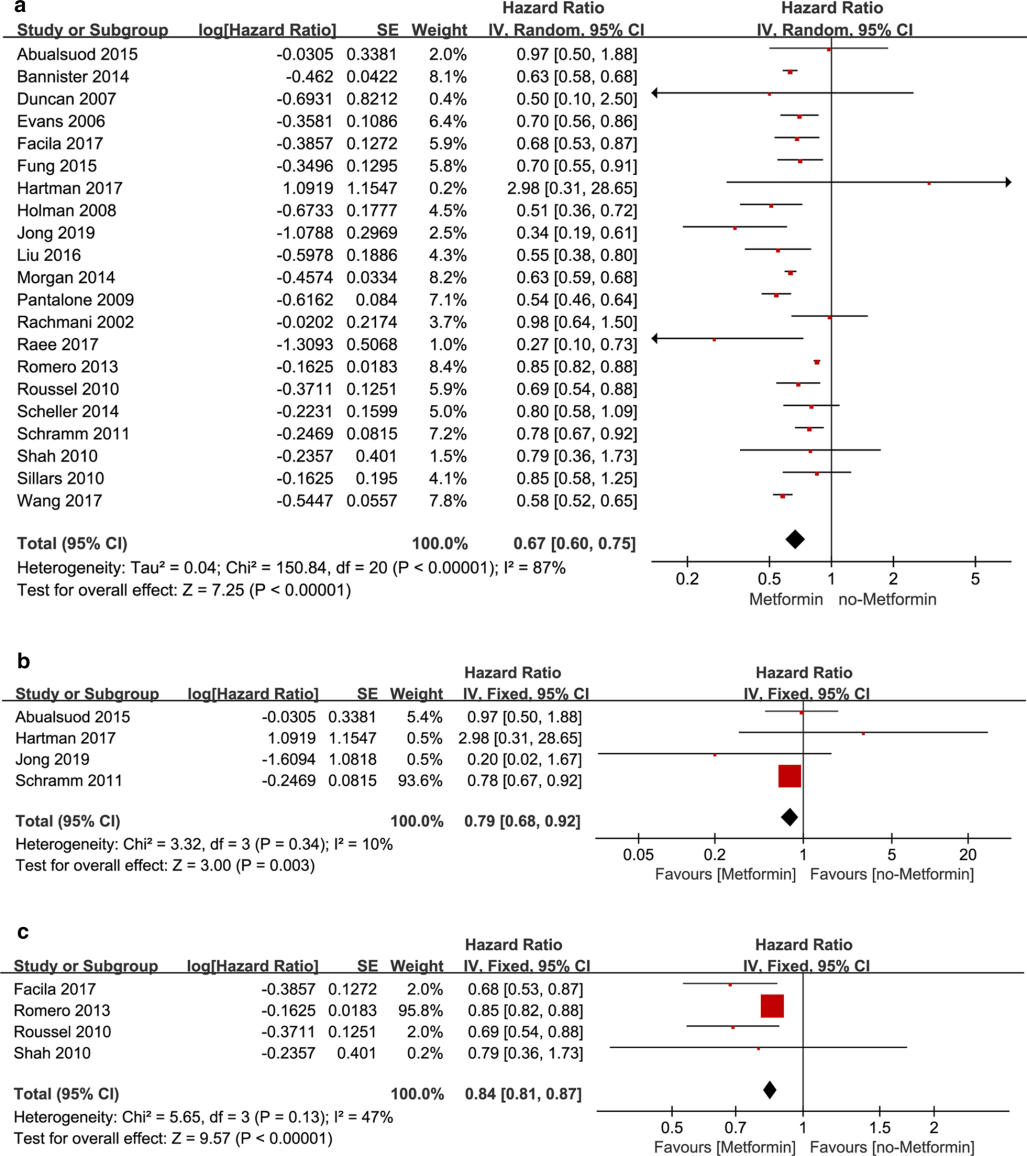
**a** Forest plot of hazard ratio of all-cause mortality among patients with metformin therapy vs no-metformin therapy. **b** Forest plot of hazard ratio of all-cause mortality among patients with MI at baseline, metformin therapy vs no-metformin therapy. **c** Forest plot of hazard ratio of all-cause mortality among patients with HF at baseline, metformin therapy vs no-metformin therapy

Secondly, we investigated the association between metformin and all-cause mortality. Out of included 21 studies only one study Hartman 2017 presented aHR > 1 [30], 20 studies presented aHR of all-cause mortality between metformin and non-metformin less than 1 (aHR < 1), the adjusted aHR is 0.67 [95% CI 0.60, 0.75] (*P* < 0.00001), heterogeneity is greater *I*^2^ = 87% (Fig. 3a). It means that metformin is helpful in lowering all-cause mortality.

Thirdly, an analysis of two subgroups was carried out. The aHR of all-cause mortality in patients diagnosed with MI according to baseline characteristics in one subgroup was 0.79 [95% CI 0.68, 0.92], (*P* = 0.003) with small heterogeneity *I*^2^ = 10% (Fig. 3b). The aHR of all-cause mortality in patients diagnosed with HF according to baseline characteristics in the other subgroup was 0.84 (95% CI 0.81, 0.87), (*P* < 0.00001), with small heterogeneity *I*^2^ = 47% (Fig. 3c). The analysis suggested that metformin could reduce all-cause mortality in patients with MI and patients with HF diagnosed according to baseline characteristics. It is worthy to note that the aHR was pooled based on the biggest adjustment of the regression model (the largest adjustment model was used to estimate the risk).

### Association of metformin with the incidence of cardiovascular events

We carried out detailed subgroup analyses of the effect of metformin on cardiovascular events, so that the pooled estimates were not affected by the heterogeneity due to the interventions and categories of patient’s baselines. Based on patient’s baseline characteristics we allocated the patients into HF subgroup and MI subgroup, type II diabetes mellitus (T2DM) subgroup and non-T2DM subgroup; based on given drugs, we allocated the patients into sulphonylurea subgroup and non-drug subgroup. We had a total of six subgroup analyses.

**Fig. 4:**
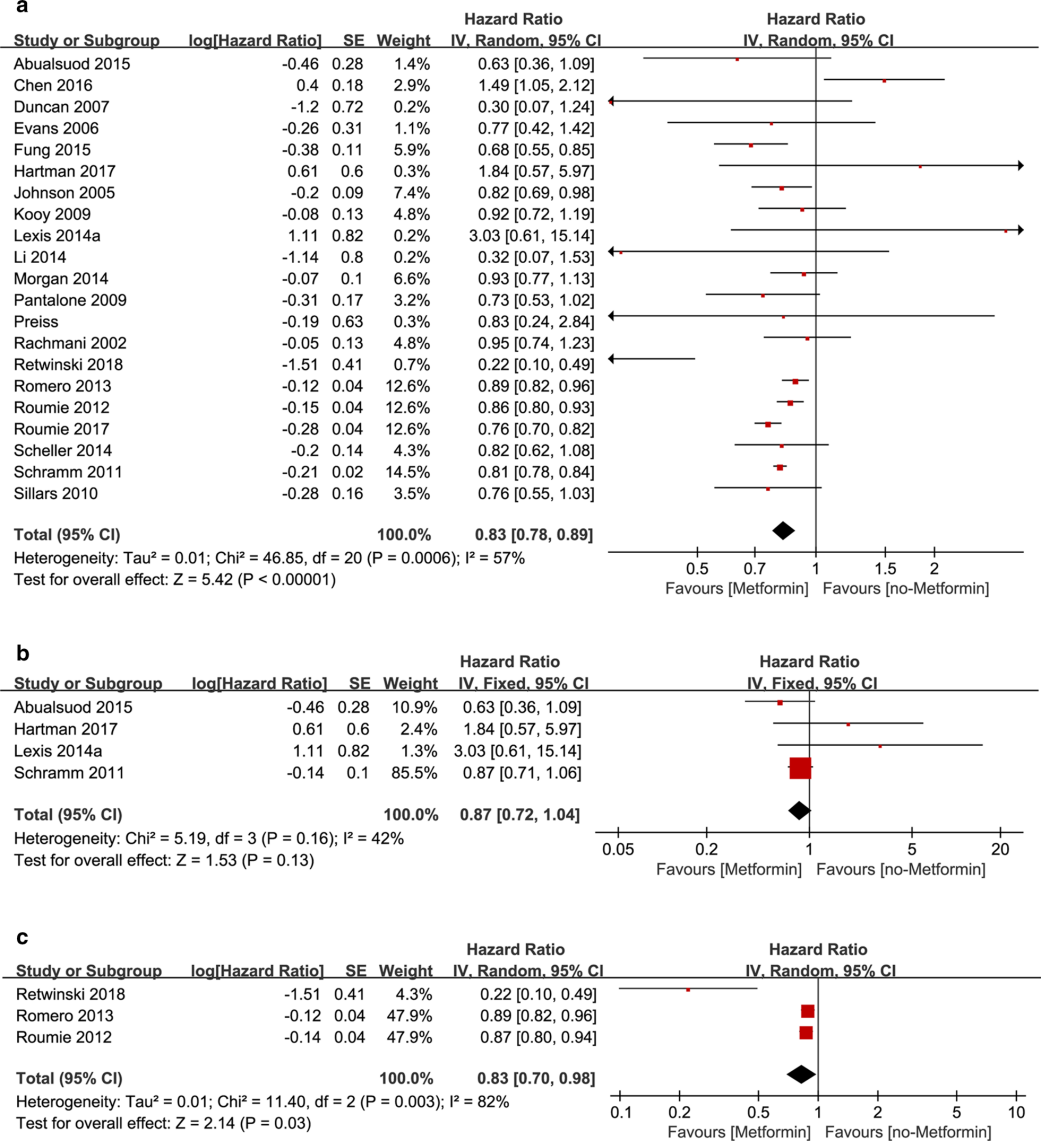
**a** Forest plot of hazard ratio of CV events among patients with metformin therapy vs no-metformin therapy. **b** Forest plot of hazard ratio of CV events among patients with MI at baseline, metformin therapy vs no-metformin therapy. **c** Forest plot of hazard ratio of CV events among patients with HF at baseline, metformin therapy vs no-metformin therapy

We included 21 studies and assessed HR of incidence of cardiovascular events between metformin trials and non-metformin trials for all patients without subclass analysis. Only two studies (Hartman [30] and Lexis [39]) presented HR > 1, all other 19 studies reported HR < 1, pooled aHR was 0. 83 (95% CI 0. 78, 0. 89), (*P* < 0.00001) (Fig. 4a). It suggested that metformin could reduce the incidence of CV events. *I*^2^ = 57%, heterogeneity was moderate and randomized effects model was used.

### CAD subgroup analysis according to patient’s baseline

In MI subgroup four studies reported the pooled aHR was 0.87 [95% CI 0.72, 1.04] (*P* = 0.13), *I*^2^ = 42% (Fig. 4b). In HF subgroup three studies reported the pooled aHR was 0.83 [95% CI 0.70, 0.98] (*P* = 0.03), I^2^ = 82% (Fig. 4c). Analysis suggested that the incidence of cardiovascular events in HF patients who took metformin was lower than those who didn’t take metformin. The pooled aHR for MI subgroup, though, less than 1, was not statistically significant, suggested that metformin had not significant effect on MI patients.

### T2DM/non-T2DM subgroup analysis according to patient’s baseline

**Fig. 5:**
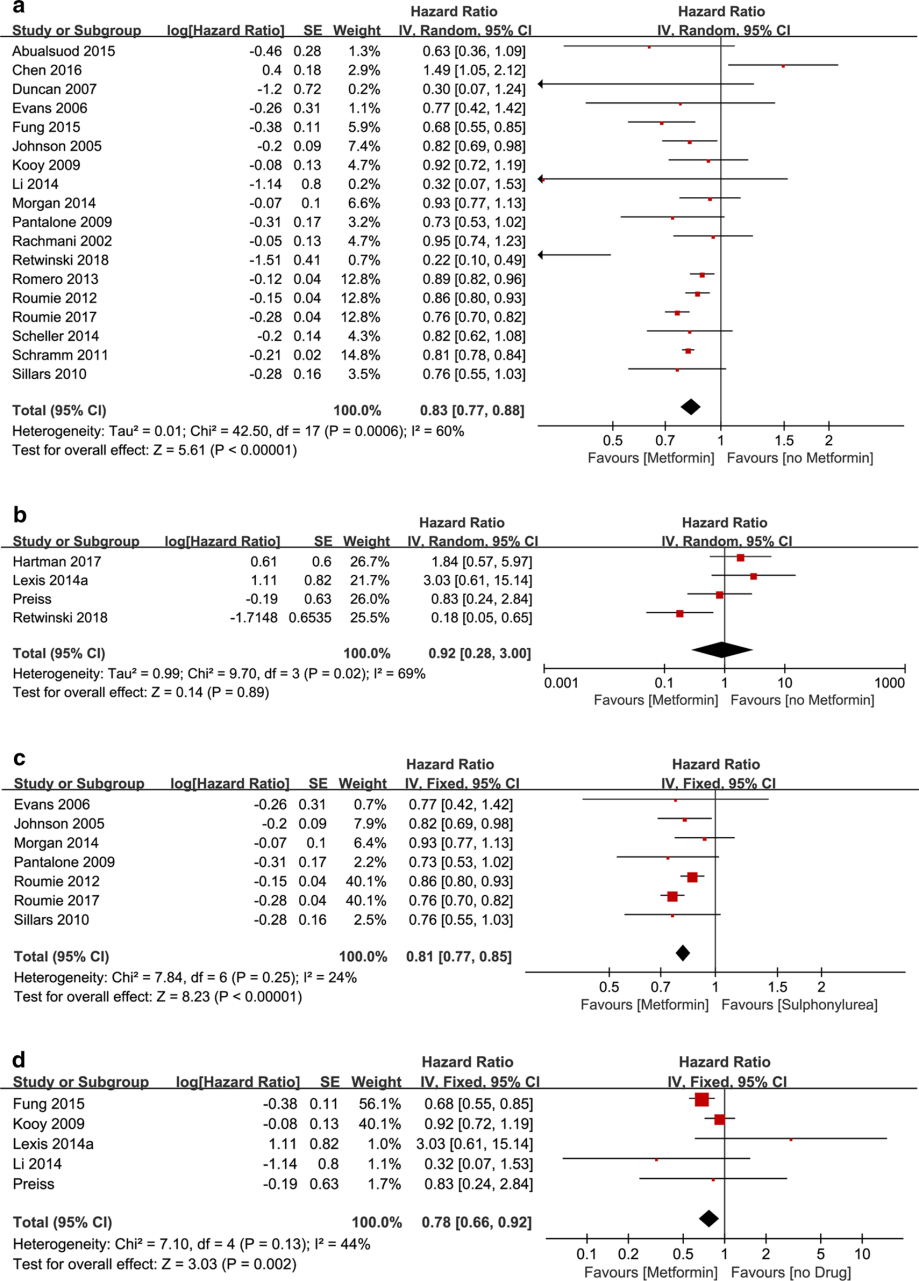
**a** Forest plot of hazard ratio of CV events VENTS among patients with T2DM at baseline, metformin therapy vs no-metformin therapy. **b** Forest plot of hazard ratio of CV events among patients without T2DM at baseline, metformin therapy vs no-metformin therapy. **c** Forest plot of hazard ratio of CV events among patients with metformin monotherapy vs sulphonylurea monotherapy. **d** Forest plot of hazard ratio of CV events among patients with metformin monotherapy vs no-drug therapy

In the T2DM subgroup, 18 studies reported the pooled aHR was 0.83 [95% CI 0.77, 0.88] (*P* < 0.00001), *I*^2^ = 60% (Fig. 5a), suggesting that the incidence of cardiovascular events in diabetic patients who took metformin was lower than those who didn’t take metformin. In non-T2DM subgroup, four studies reported the pooled aHR was 0.92 [95% CI 0.28, 3.00] (*P* = 0.89), *I*^2^ = 69% (Fig. 5b). The pooled aHR, though less than 1, was not statistically significant, suggested that metformin had no significant effect on non-diabetic patients.

### Drugs subgroup analysis

In sulphonylurea subgroup, on the subject of incidence of cardiovascular events in metformin trials and sulphonylurea trials, seven studies reported the pooled aHR was 0.81 [95% CI 0.77, 0.85] (*P* < 0.00001), *I*^2^ = 24% (Fig. 5c), suggesting that metformin was more helpful than sulphonylurea in reducing the incidence of cardiovascular events. In non-drug subgroup five studies reported the pooled aHR was 0.78 [0.66, 0.92] (*P* = 0.002), *I*^2^ = 44% (Fig. 5d), suggesting that metformin was more helpful than non-medication in reducing the incidence of cardiovascular events.

### Association of metformin with cardiac function

**Fig. 6:**
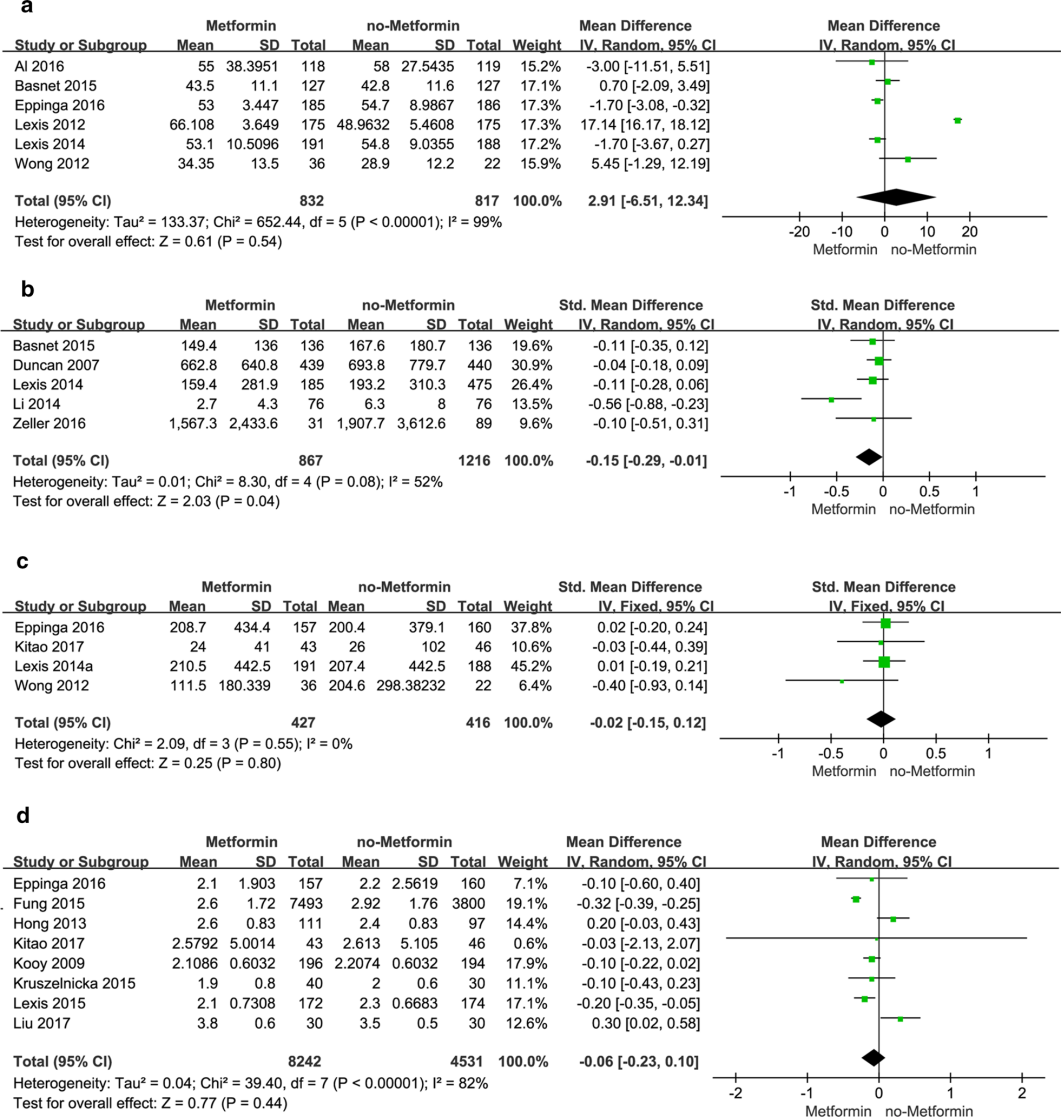
**a** Forest plot of mean difference of LVEF% among patients with metformin therapy vs no-metformin therapy. **b** Forest plot of mean difference of CK-MB among patients with metformin therapy vs no-metformin therapy. **c** Forest plot of mean difference of BNP among patients with metformin therapy vs no-metformin therapy. **d** Forest plot of mean difference of LDL among patients with metformin therapy vs no-metformin therapy

The effect of metformin on the cardiac function was discussed in our studies. Left ventricular ejection fraction (LVEF) is the key indicator of cardiac functions. Anything less than 50% of LVEF indicates a serious impairment of cardiac ejection. In this comparison, LVEF was investigated in six studies: three studies showed that the mean value of LVEF in the metformin trials was higher than that in the control trials, while the other three studies showed the opposite. LVEF in the metformin trials vs the non-metformin trials (MD 2.91; 95% CI − 6.51 to − 12.34) (Fig. 6a). Although LVEF in the metformin trials was higher than that in the non-metformin trials, the pooled values (*P* = 0.54) were not statistically significant. As heterogeneity was large (*I*^2^ = 99%), the inverse variance approach and random-effects models were used in this meta-analysis.

Creatine kinase MB (CK-MB) is mainly found in myocardial tissue. It is an important indicator of acute myocardial impairment and is often used as an auxiliary diagnostic tool for acute myocardial infarction (AMI). An analysis was then carried out to explore the effect of metformin on the reduction of CK-MB. In this comparison, we reviewed five studies, all of which observed that the mean value of CK-MB in the metformin trials was lower than that in the non-metformin trials, but four studies had no statistical significance. The pooled standard mean difference (SMD) of CK-MB is − 0.15 [− 0.29, − 0.01], (*P* = 0.04); *I*^2^ = 52%, suggesting that metformin could reduce CK-MB (Fig. 6b).

Type B natriuretic peptide (BNP) is a widely used biomarker for cardiac function and is mainly found in heart ventricle. Increased ventricular volume and pressure can lead to increased plasma BNP, which reflects the change of left ventricular function. BNP is often used to assist in the diagnosis of heart failure and to determine the severity and prognosis of the condition. So, we analyzed the effect of metformin on BNP. four studies were included in the BNP comparison. 3 studies observed the mean value of BNP in the metformin group was lower than that in the control group. 1 study found the opposite. This comparison revealed insignificant result (SMD − 0.02; 95% CI 0.15–0.12; *P* = 0.8) (Fig. 6c). The heterogeneity was 0 (*I*^2^ = 0%), a fixed effect model was used. 4 studies, though had different measures of effect indexes, all pointed to the same cardiac function, so SMD was used instead of MD.

### Association of metformin with LDL level

Hyperlipidemia, especially high LDL, are important risk factors for cardiovascular and cerebrovascular diseases. The reduction of LDL has been shown to reduce cardiovascular risk and mortality. 8 studies were included in this paper on the relationship between metformin and LDL. 1 study revealed statistical significance, while six studies reported that mean value of LDL in metformin group was lower than that in control group. Although the pooled effect size revealed that the level of LDL in metformin trials was lower than that in non-metformin trials, the pooled effect size was not statistically significant (MD − 0.06 [− 0.23, 0.10]; *I*^2^ = 82%; *P* = 0.44 (Fig. 6d).

### Risk of bias across studies

**Fig. 7:**
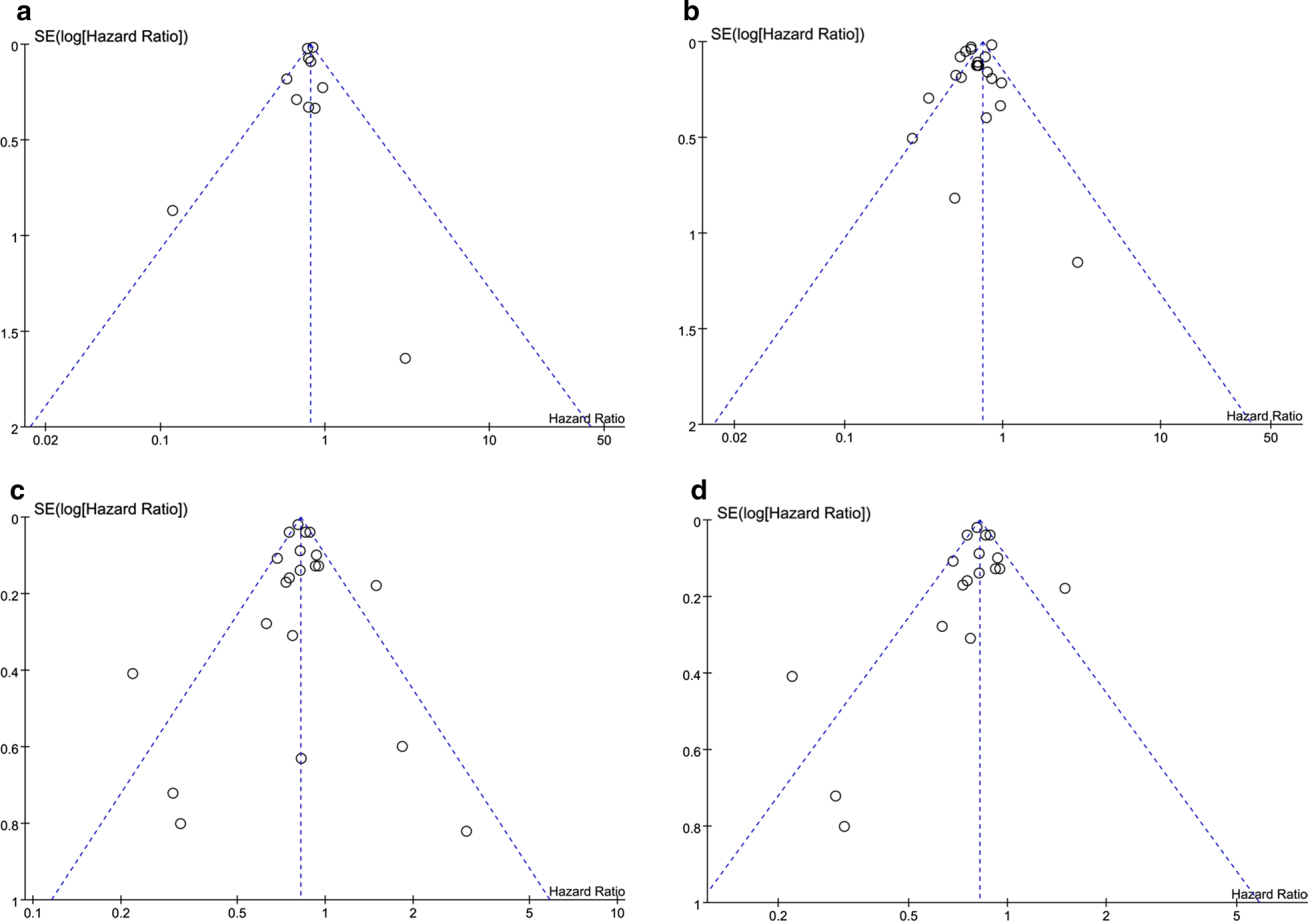
**a** Funnel plot of hazard ratio of cardiovascular mortality among patients with metformin therapy vs no-metformin therapy. **b** Funnel plot of hazard ratio of all-cause mortality among patients metformin therapy vs no-metformin therapy. **c** Funnel plot of hazard ratio of CV events among patients with metformin therapy vs no-metformin therapy. **d** Funnel plot of hazard ratio of CV events among patients with T2DM at baseline, metformin therapy vs no-metformin therapy

The funnel plots of Review Manager 5.3 give us a visual assessment of the publication bias of four comparisons: HR of cardiovascular mortality (Fig. 7a), HR of all-cause mortality (Fig. 7b), HR of incidence of cardiovascular events (Fig. 7c) and HR of incidence of cardiovascular events in subgroup of diabetic patients (Fig. 7d). You can tell from the four symmetrical figures that the publication bias isn’t large. Funnel plot is useless for other comparisons because of less than 10 studies included.

### Sensitivity analysis

Sensitivity analysis was performed for three comparisons of cardiac function and one comparison of LDL, by sequentially removing one study at a time and observing the exaggerated effect this had on the overall result. Removing anyone study didn’t have any effect on overall result in three comparisons of BNP, LDL and LVEF. But in comparison of CK-MB, removing anyone (apart from Duncan [25]) would lead to changes to pooled value and changes into statistical insignificance, that means this comparison requires more studies to draw more reliable conclusions.

## Discussion

40 clinical trials were included in this study involving 1,066,408 subjects. We found: (1) metformin could remarkably reduce cardiovascular mortality; (2) metformin could significantly reduce all-cause mortality, including in patients with MI and HF; (3) metformin could reduce the incidence of cardiovascular events. Metformin could significantly reduce the incidence of cardiovascular events in HF patients, but wasn’t effective in MI patients. Metformin could significantly reduce the incidence of cardiovascular events in T2MD patients, but wasn’t effective in patients without T2MD. Furthermore, metformin was effective in reducing the incidence of cardiovascular events compared to those who take sulfonylureas or those who didn’t take anything; (4) metformin could reduce CK-MB level, but couldn’t improve LEVF and BNP; (5) metformin couldn’t lower LDL levels.

Metformin is the most widely used oral antihyperglycemic agent for the treatment of type 2 diabetes. Twenty years ago, experimental evidence showed that lowering blood glucose reduced the risk of microvascular complications among patients with type 2 diabetes [33, 60, 61]. Several studies have subsequently indicated that metformin decreased mortality and adverse cardiovascular events [62–66]. A large nationwide study revealed that patients treated with metformin had significantly lower all-cause mortality than those treated with sulfonylureas (including glimepiride, glyburide, glipizide, and tolbutamide) [54]. A retrospective cohort study of 23,915 patients with type 2 diabetes mellitus reported that patients treated with glipizide [67], glibenclamide or glimepiride had higher mortality than those treated with metformin after a median follow-up of 2.2 years. However, Bannister et al. [68] found that the survival rate of diabetic patients using metformin was almost the same as that of normal healthy people. Inzucchi et al. [69] also found that the mortality risk of patients using metformin at discharge was similar to that of patients not using metformin. Therefore, the cardiovascular protective effect of metformin was not clear. This meta-analysis indicates that metformin can reduce the cardiovascular mortality, all-cause mortality and incidence of cardiovascular events. In order to make the discussion more accurate, we conducted subgroup analyses. We find that metformin can reduce the incidence of cardiovascular events not only in HF patients, but also in T2MD patients, though, it isn’t effective in MI patients and patients without T2DM. In addition, metformin reduces the incidence of cardiovascular events better than sulfonylureas and better than non-medication. Metformin can significantly reduce mortality and reduce the incidence of cardiovascular events, that means metformin can be used as a cardiovascular protective agent to prolong life and reduce mortality. However, the cardiovascular protective mechanism of metformin has not been fully elucidated. T2DM is often associated with cardiovascular complications, and the two often coexist as comorbidity. Patients with T2DM often have elevated levels of inflammatory cytokines, while hyperglycemia and high concentration of glycation end products can also cause vascular endothelial cell damage and calcification [70], which have important effects on the onset and progression of atherosclerosis [61, 71], leading to related cardiovascular diseases. Metformin activates AMPK phosphorylation, which reduces oxidative stress, reduces the production of inflammatory cytokines, and increases eNOS (endothelial nitric oxide synthase) activity, which may be an important mechanism for metformin cardiovascular protection. Recent studies have also shown that metformin has protective effects on vascular endothelial function and angiogenesis [72], which may be the pathway of metformin’s cardiovascular protective effects. However, this protective effect also has some limitations. For patients with severe diseases, such as MI, metformin has no effect, nor does it have cardiovascular protective effect for CAD patients without diabetes. Therefore, metformin cannot be used as first-line cardiovascular therapy. However, metformin can effectively reduce all-cause mortality, cardiovascular mortality and the incidence of cardiovascular events in patients with T2DM. Based on this cardiovascular protective effect, it is recommended that patients with T2DM should be given priority in the use of metformin, which can reduce the incidence of cardiovascular events while treating diabetes. Even if patients with T2DM have already had cardiovascular complications, it is recommended to use metformin in combination with cardiovascular therapy.

In diabetic patients, metformin and improved prognosis are independent of glycemic control, and there are indications that it may have direct cardioprotective effects [37, 73]. Metformin has also been reported to improve cardiac cell function by altering cardiac metabolism and remodeling. Studies in mice found metformin reduced infarct size by 22 to 65%. Long-term preoperative use of metformin can reduce the risk of non-infarction in patients with acute myocardial infarction [74]. A 2014 retrospective cohort study found that metformin also reduced the size of myocardial infarction [37]. However, metformin’s role in improving cardiac function remains controversial. In one study, while patients with acute st-segment elevation patients (STEMI) were treated with metformin for 4 months, metformin was not found to increase ejection fraction (53.1% vs 54.8%, *P* = 0.1) or decrease n-terminal pro-brain natriuretic peptide (nt-probnp) levels (167 vs. 167 ng/L, *P* = 0.66) [39]. Another STEMI study also found no improvement in ejection fraction in diabetic patients treated with metformin and a 0.7% difference between those taking metformin and those not taking metformin [23]. After it was shown it could reduce mortality, metformin was further analyzed in this meta-analysis for its effect on cardiac function: LVEF, CK-MB and BNP were specifically analyzed. Our analysis revealed that the use of metformin is not associated with increased LVEF and decreased BNP, but with decreased mean value of CK-MB. In short, we found that metformin may have some effects on cardiac function. However, due to the small number of studies on CK-MB, the bias could be very large and there might be errors. More studies are needed to confirm the effect of metformin on CK-MB.

Hyperlipidemia is an important risk factor for coronary heart disease [75]. For every 1.0 mmol/L increase in LDL, the risk of acute cardiovascular events increases by about 40%. Lowering blood lipids can effectively reduce the incidence of coronary heart disease [1, 2]. Previous studies have shown that metformin can alter hepatic lipid homeostasis by inhibiting acetyl CoA carboxylase by AMPK, thereby enhancing insulin action. The level of total blood cholesterol and LDL can be reduced by taking metformin in the elderly [76]. The plasma total cholesterol (TG) is remarkably reduced by using high dose metformin (> 1700 mg/day). Lexis et al. [38] found that LDL levels in non-diabetic acute STEMI patients significantly decreased after 4 months of administration of metformin. These studies show that metformin also lowers blood lipid levels. For further assessment of the effect of metformin on LDL levels nine studies were included in this meta-analysis, though six studies showed that metformin reduced LDL levels, our meta-analysis found that metformin was not associated with reduced LDL levels. Luo et al. [62] also concluded that metformin does not reduce LDL concentration, but it plays a cardiovascular protective role by increasing cholesterol outflow [17]. So, more research is needed on whether metformin may be protective by affecting LDL.

As a hypoglycemic drug, metformin has been used clinically for up to 60 years thanks to its low cost, safety and effectiveness. In our meta-analysis, it was found that besides the antihyperglycaemic effect, metformin can also reduce all-cause mortality, mortality of cardiovascular disease and incidence of CV events. Besides the antihypoglycemic effect, clinicians should consider metformin’s cardiovascular protection when selecting drugs. Metformin is more secure because it has a very low risk of hypoglycemia compared to other drugs such as sulfonylureas, and very little risk of causing lactic acidosis. We have more sufficient reasons to recommend metformin.

Some limitations should be considered when interpreting our findings. First of all, most of the patients included in this study were diabetic, so the results do not stand for the majority of non-diabetic patients with coronary heart disease treated with metformin. Secondly, there is large heterogeneity between some studies related to population, research design and other aspects. Thirdly, this meta-analysis doesn’t reflect other language studies as literature included is written in English.

## Conclusions

In conclusion, this meta-analysis shows that metformin can significantly reduce cardiovascular mortality and all-cause mortality as well as the incidence of cardiovascular events in diabetic and non-diabetic patients with coronary heart disease. Metformin has no significant effect of reducing the incidence of cardiovascular events in MI patients and CAD patients without T2DM. Furthermore, metformin has a better effect of reducing the incidence of cardiovascular events than sulfonylureas and non-medication. This meta-analysis provides new ideas for doctors to choose hypoglycemic drugs.

## Data Availability

Not applicable.

## Abbreviations

aHR: adjusted hazard ratio
BNP: type B natriuretic peptide
CAD: coronary artery disease
CI: confidence interval
CK-MB: creatine kinase MB
CV events: cardiovascular events
HF: heart failure
HR: hazard ratio
LDL: low density lipoprotein
LVEF: left ventricular ejection fraction
MI: myocardial infarction
NOS: Newcastle–Ottawa Scale
SMD: standard mean difference
T2DM: type II diabetes mellitus
